# Secret of Eternal Youth; Teaching from the Centenarian Hot Spots (“Blue Zones”)

**DOI:** 10.4103/0970-0218.58380

**Published:** 2009-10

**Authors:** Badri N Mishra

**Affiliations:** Prof of Community Medicine, Rural Medical College, PIMS, Loni. Maharastra, India

The euphoria of modern day health achievements is in an epidemiological cross road. We are going to lose out the hard caught health benefits to modern monsters such as obesity and its associated diseases. For example, the present American life expectancy of 77.8 years is expected to plummet due to the current trend of noncommunicable diseases.(1) However, there are zones (blue zones) on our planet where people live like in fairy tales, holding the secret of eternal youth.(2) Time has come to explore these areas to enrich our academic and research.

Scientific exploration has shown some basic factors as being behind a long lifespan: a plant-based diet; regular, low-intensity activity; an investment in family; a sense of faith; and purpose.(3–8)

## Blue Zones

Okinawa Island in Japan is famous for the longest disability-free life expectancy in the world.(4–10) Other “blue zones” are Sardinia (Italy) with the highest concentration of centenarians and the community of Seventh Day Adventists of Loma Linda in California. Nicoya Peninsula in Costa Rica is another “blue zone” where healthy centenarians live in a solid support network of friends and family.(6)

People who make it to a hundred tend to be nice. They drink from the fountain of life by being likable and drawing people to them. As an example, Nicoya Peninsula has the lowest middle-age mortality in the world. Here a 60-year-old has more than fourfold better chance of making it to 90 than a 60-year-old in America.(7) They spend 20% the amount on public health in contrast to American, but they spend it in the right places.(6)

So set up your life, your home environment, your social environment, and your workplace so that you are constantly nudged into behaviors that favor longevity. Let us look at the blue zone in Okinawa, these people are consistently eating off of small plates. One of the cues for fullness is an empty plate, so stock your cupboard with smaller plates. Add decades to your life by reconnecting with your religion and investing in your family.(6–10)

## The Okinawa Case

Okinawa, the main island of Ryukyu in Japan, is packed with natural adversities. It witnessed a fierce and crucial battle during World War II. Today, the US military bases occupy 20% of its surface and the Okinawa Prefecture is the poorest province of Japan.(9)

In contradiction to the above facts, the heaven of longevity is here. Besides holding the record for longevity and centenaries of the world, it is the only place where 80-90 year-olds live like persons 30 years younger. Ancient Chinese legends already called Okinawa “the land of the immortals”. Here you see 100-year-old persons that are not even thinking about retirement. The prevalence of centenarians is of 35 for each 100 000 inhabitants.(8–11)

The Okinawa inhabitants reach ages similar to the Japanese average of 86 for women and 78 for men.(9) However, the real encouraging factor is not that people reach these ages, but they grow old in a much better state. The statistics reveal a significantly lower risk of heart attack and stroke, cancer, osteoporosis, Alzheimer. Some in their 90's can honestly vouch that they still have an active sex life.(11)

Research show that there are two main factors behind the longevity on this island: alimentation and a healthy lifestyle that experts believe can be imitated.

## Okinawa Diet

Vegetables grown on this volcanic soil are rich in vitamin C and polyphenol, which have antioxidant functions. They are a great source of dietary fiber and potassium. People eat 300 g of vegetables a day. The increased consumption of yellow-green Okinawa vegetables that include soybeans, tofu and goya - a bitter gourd improves biological health indices. Older Okinawans eat probably three or four times more of vegetables than that of the younger generations.(9)

Okinawan women also have a very high intake of natural estrogens through their diet, mainly from the soy they consume. Soy contains phytoestrogens, or plant estrogens called flavonoids. The other important major phytoestrogens are lignans, which are derived from flax and other grains. All plants, especially legumes, onions, and broccoli, contain these natural estrogens, but not nearly in the same quantity as soy and flax. Recent double-blind placebo controlled studies support the ability of soy isoflavones to slow the bone loss that occurs with menopause.(11)

Elderly were found to have impressively young, clean arteries, low cholesterol, and low homocysteine levels when compared to Westerners. They have low blood levels of free radicals. The elders had significantly lower levels of lipid peroxide-compelling evidence that they suffer less free-radical-induced damage.(11) These factors help reduce their risk for coronary heart disease by up to 80% and keep stroke levels low.

Okinawan centenarians have been lean throughout with an average body mass index (BMI) of 18 to 22. They traditionally keep eating a low-calorie, low glycemic load diet, practice calorie control in a cultural habit known as hara hachi bu (only eating until they are 80% full), and staying physically active the natural way.(11)

## Okinawa Social Support

Nowhere is a strong sense of purpose more acute than in Japan, where the concept has its own name: “ikigai' (You see it over and over again).(4) The elders here have low depression levels. You can see persons aged 90-100 on motorcycles or mountain bikes on the streets. Most of them practice karate, kendo, dancing, walk daily several kilometers, and even work on vegetable gardens and after that sell the products.

These people do not go to fitness gyms or do jogging; they can, however, practice profound respiration, tai-chi (a type of yoga), and gardening and other activities in the open that positively influence the stress level.

They are preoccupied by hobbies, linked to a social network that makes them feel connected to the environment and their fellows, and helps develop their spiritual side. Physical activity is not isolated, but has an objective, making the elders feel active members of the community.

Another factor in Okinawa is that people take care of each other, forming more coherent and supportive links than in the western world. They have a positive attitude toward life which explains the extremely low levels of stress experienced by these elders. Of course, there is a genetic factor that may also be contributing to this vitality.

## Longevity Gene

The key to a long life is now believed to be a mix of genetics and environmental factors such as health habits and stress free events.(10)

While talking about gene, it is worth mentioning the effort of the New England Centenarian Study at Boston University. It has gathered DNA information from about 100 super centenarians (people who live to 110 and beyond). Study suggests that several alleles of the HLA-DRB1 and/or HLA-DQ genes are involved in human longevity.(10) The analysis of HLA class II genes of Okinawan centenarians using the polymerase chain reaction-restriction fragment length polymorphism (PCR-RFLP) method yielded that the frequencies of DQB1*0503, DQA1*0101 (04) and DQA1*05 were increased in the centenarians, whereas those of DQA1*0102, DQA1*0103 and DQB1*0604 were decreased. Similarly, for the DRB1 gene, the frequencies of DRB1*0101, DRB1*1201 and DRB1*1401 were increased in the centenarians, whereas those of DRB1*0403 and DRB1*1302 were decreased.

## Conclusion

Although the blue zones are found in four very geographically and culturally different parts of the world, there are nine characteristics common to all of them that are portable to any location and can be used to make healthy lifestyle changes. They include making low-intensity physical activity part of one's daily routine, building good relationships with friends and family, eating a diet lighter on meat and excess calories and heavier on plants, and finding a purpose for and sense of meaning in your life.

You can optimize your lifestyle, you may gain back an extra decade of good life you'd otherwise miss. Adopting any one of these nine will immediately improve your life expectancy at any age.
